# Associations between health systems capacity and mother-to-child HIV prevention program outcomes in Zambia

**DOI:** 10.1371/journal.pone.0202889

**Published:** 2018-09-07

**Authors:** Joan T. Price, Benjamin H. Chi, Winifreda M. Phiri, Helen Ayles, Namwinga Chintu, Roma Chilengi, Jeffrey S. A. Stringer, Wilbroad Mutale

**Affiliations:** 1 School of Medicine, University of North Carolina at Chapel Hill, Chapel Hill, North Carolina, United States of America; 2 UNC Global Projects Zambia, Lusaka, Zambia; 3 Zambart, Lusaka, Zambia; 4 Society for Family Health, Lusaka, Zambia; 5 Center for Infectious Disease Research in Zambia, Lusaka, Zambia; 6 University of Zambia School of Public Health, Lusaka, Zambia; The Ohio State University, UNITED STATES

## Abstract

**Introduction:**

Zambia has made substantial investments in health systems capacity, yet it remains unclear whether improved service quality improves outcomes. We investigated the association between health system capacity and use of prevention of mother-to-child HIV transmission (PMTCT) services in Zambia.

**Materials and methods:**

We analyzed data from two studies conducted in rural and semi-urban Lusaka Province in 2014–2015. Health system capacity, our primary exposure, was measured with a validated balanced scorecard approach. Based on WHO building blocks for health systems strengthening, we derived overall and domain-specific facility scores (range: 0–100), with higher scores indicating greater capacity. Our outcome, community-level maternal antiretroviral drug use at 12 months postpartum, was measured via self-report in a large cohort study evaluating PMTCT program impact. Associations between health systems capacity and our outcome were analyzed via linear regression.

**Results:**

Among 29 facilities, median overall facility score was 72 (IQR:67–74). Median domain scores were: patient satisfaction 75 (IQR 71–78); human resources 85 (IQR:63–87); finance 50 (IQR:50–67); governance 82 (IQR:74–91); service capacity 77 (IQR:68–79); service provision 60 (IQR:52–76). Our programmatic outcome was measured from 804 HIV-infected mothers. Median community-level antiretroviral use at 12 months was 81% (IQR:69–89%). Patient satisfaction was the only domain score significantly associated with 12-month maternal antiretroviral use (β:0.22; p = 0.02). When we excluded the human resources and finance domains, we found a positive association between composite 4-domain facility score and 12-month maternal antiretroviral use in peri-urban but not rural facilities.

**Conclusions:**

In these Zambian health facilities, patient satisfaction was positively associated with maternal antiretroviral 12 months postpartum. The association between overall health system capacity and maternal antiretroviral drug use was stronger in peri-urban versus rural facilities. Additional work is needed to guide strategic investments for improved outcomes in HIV and broader maternal-child health region-wide.

## Background

Over the last decade, tremendous gains have been made in pediatric HIV, largely through the implementation of effective interventions for the prevention of mother-to-child HIV transmission (PMTCT)[1]. Strong scientific evidence and forward-thinking policies have led to the widespread uptake of antiretroviral therapy (ART) by pregnant and breastfeeding women globally[2, 3]. Increasing coverage of PMTCT programs in priority countries has brought about a 60% global decline in new pediatric HIV infections since 2010[4], with most countries reporting significant reductions in mother-to-child HIV transmission and some achieving near elimination[5, 6].

Although a number of factors may influence access to PMTCT services, the capacity of health systems to meet demand is critical and may serve as important determinants for uptake, adherence, and retention[7]. A range of health systems attributes have been associated with program outcomes, including service coverage and quality, care coordination and support, and clinical capacity[8–10]. While the World Health Organization (WHO) has emphasized the need for health system strengthening[11], there remains scarce evidence of how strategic interventions to improve health system capacity may translate into greater service coverage, decreased attrition, and improved patient-level outcomes.

In this study, we sought to investigate this possible relationship in the context of PMTCT services in Zambia’s Lusaka Province. Leveraging data from two parallel, related evaluations, we focused on a single, important programmatic outcome: maternal antiretroviral use at 12 months following delivery. As PMTCT programs continue to expand globally, there is need to identify those health systems factors that have greatest impact on health outcomes.

## Materials and methods

For this secondary analysis, we used data from two related studies conducted between 2014 and 2015 in rural and semi-urban Lusaka Province, Zambia. Health systems capacity was assessed through the Better Health Outcomes through Mentoring and Assessment (BHOMA) initiative, which sought to improve the quality of primary care through community- and facility-based interventions[12]. Our outcome data were obtained from the community cohort component of the Survey Validation Project (SUVA), a validation of a household survey methodology for evaluating population-level PMTCT effectiveness. The community cohort served as the “gold standard” for measuring the primary outcome: HIV-free survival among infants born to known HIV-infected women[13].

### Study setting

The BHOMA and SUVA studies took place across an extensive network of government clinics and hospitals that comprise essentially all public sector facilities within four of the five districts in Lusaka Province, Zambia: Chilanga, Chongwe, Kafue, and Rufunsa. The studies were not implemented in urban Lusaka District. The combined population of these four districts was approximately 306,000 at the time of study implementation. Prevalence of HIV among all adult women in Lusaka Province is 19.4%, while prevalence in the rural and peri-urban areas is usually slightly lower[14]. At the time of the studies’ implementation, the Zambian Ministry of Health had recently adopted the WHO-recommended “Option B+” approach of lifelong ART for all HIV-infected pregnant women, a policy that evolved to become standard of care[15]. In Zambia, PMTCT programs are typically integrated into maternal-child health services including postnatal care.

### Health systems capacity assessment: The BHOMA study

As part of the BHOMA initiative, three health facility surveys were conducted between 2011 and 2015 to assess changes in health systems capacity. In this analysis, we used data from the third and final survey, which coincided with enrollment for the SUVA community cohort (see below). Using a “balanced scorecard” approach[16], we evaluated six domains derived from the WHO building blocks for health systems strengthening: patient satisfaction, human resources, finance, governance, service capacity, and service provision[16, 17]. All study tools were based on direct observation or interviewer-administered questionnaires, except for the governance assessment which participating stakeholders completed on their own. Depending on the domain assessed, questionnaires were completed by health facility managers, health workers or patients.

The patient satisfaction domain comprised adult satisfaction and child satisfaction indices, which were assessed by exit interviews among five adults and five under-five child/guardian pairs, respectively[16, 18]. Interviewers asked participants to rate each of the following on a 5-point scale: wait time to be seen by a provider, the provider’s explanation of illness and treatment, and the perceived appropriateness of the treatment received. The human resources domain was assessed by two indices: the health worker motivation score (a composite of 23 items affecting motivation[19]) and the proportion of interviewed health workers who had received job training in the preceding 12 months. The finance domain was calculated based on availability of a financial action plan, availability of a person in charge of finance at the facility, and whether the person in charge had received job training in the preceding 12 months. The governance domain was assessed by a 16-item questionnaire self-administered by a team of managers and lay workers at each facility. This tool was adapted and validated in Zambia using methodology described elsewhere[20]. The service capacity domain comprised indices of basic infrastructure, basic equipment, laboratory capacity, drug availability, and infection control. The service provision domain included indices of service readiness, adult clinical observation and pediatric clinical observation. The service readiness index was assessed by a facility audit of availability of selected essential health services, existence of guidelines and protocols, and recent use of service registers. Clinical observation indices were based on five adult encounters (irrespective of presenting complaint) and five pediatric encounters of children less than five years of age presenting with fever, cough, or diarrhea.

### Programmatic outcome

In the SUVA cohort, HIV-infected women and their HIV-exposed newborns (≤30 days of life) were recruited at the health facility and via the community-based networks from the BHOMA project[13]. Maternal HIV infection was confirmed through review of medical records (e.g., antenatal card) or through on-site rapid HIV antibody testing by trained personnel. As described previously, according to the standard of care at the time, all HIV-infected pregnant and breastfeeding women were routinely offered lifelong antiretroviral treatment. We collected demographic and medical information at enrollment, 6 weeks, 6 months, 12 months, and 18 months. Although the primary outcome was infant HIV infection or death, we also collected data about maternal antiretroviral drug use, the primary outcome for the current analysis, by asking participants: “Are you taking antiretroviral drugs now?” This information was recorded at enrollment and each subsequent study visit. Our sample size for each site was 40 mother-infant pairs. Because of a slower-than-anticipated pace of enrollment, particularly in rural sites, we later truncated follow-up to 12 months to increase the time for recruitment.

### Ethical considerations

The University of Zambia Biomedical Research Ethics Committee (Lusaka, Zambia) and the University of North Carolina Institutional Review Board (Chapel Hill, NC, USA) reviewed and approved both studies. All participants were informed about the purpose of the studies and provided written informed consent prior to taking part.

### Statistical analysis

As part of the BHOMA initiative, we conducted evaluations of health systems capacity across 42 sites in Lusaka Province. All health facilities were classified as either rural or peri-urban, as defined in the primary BHOMA study[16]. The SUVA study recruited participants from 33 of these health facilities and surrounding communities. We restricted the health facility analysis to the facilities for which we also had SUVA outcome data with sufficient sample size. We excluded four additional sites because of the small number of SUVA enrollments (<10 mother-infant pairs), out of concerns around estimate precision. Therefore, the final sample size for this secondary analysis was 29 sites. All participated in the SUVA and BHOMA studies.

Our unit of analysis was the health facility and its surrounding community (also referred to as the “site”). Health system capacity, our primary exposure, was evaluated using the balanced scorecard instrument described above[16, 18]. Domain-specific scores were calculated for patient satisfaction, human resources, finance, governance, service capacity, and service provision. We generated the overall facility score by calculating the unweighted mean of the individual indices described above. Both domain and overall facility scores could range from 0 to 100, with higher scores indicating greater facility capacity. Median overall and domain scores were calculated for all facilities and stratified by district. Variance in overall facility scores by district was investigated via the Kruskal-Wallis test by ranks.

Our primary outcome was self-reported maternal antiretroviral drug use at 12 months, a metric of health service utilization obtained from the SUVA study. We estimated an overall proportion and site-specific proportions of maternal antiretroviral drug use. To determine the possible association between health system capacity (primary exposure) and antiretroviral drug use (primary outcome), we constructed linear regression models. In our analyses, we considered the overall facility score for health systems capacity, as well as domain-specific scores. Beta coefficients of the regression, representing the change in facility-level proportion of maternal antiretroviral drug use per unit increase in facility capacity score, were estimated with 95% confidence intervals and statistical significance was defined as a *p* value of <0.05. All statistical analyses were performed using Stata version 14.1 (College Station, TX, USA).

## Results

**Fig 1:**
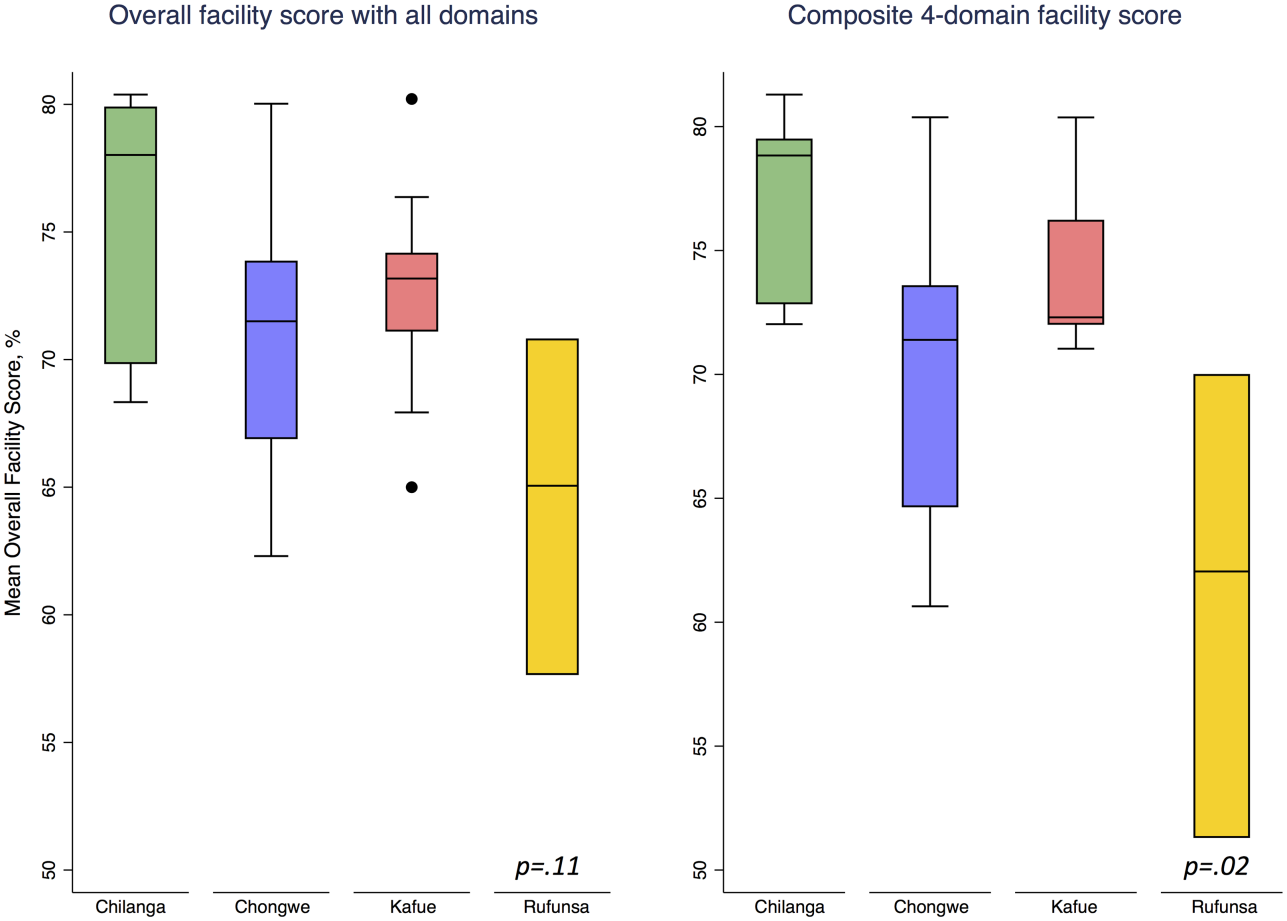
**1a**. Boxplot of median overall facility score (all domains) by district, N = 29. **1b**. Boxplot of median composite facility score (4 domains) by district, N = 29.

From June 2015 to December 2015, we evaluated health systems capacity in 42 Lusaka Province facilities. Among the 29 facilities included in this analysis, median scores calculated for each domain were as follows: patient satisfaction 75 (IQR 71–78), human resources 85 (IQR 63–87), finance 50 (IQR 50–67), governance 82 (IQR 74–91), service capacity 77 (IQR 68–79), and service provision 60 (IQR 52–76). The median overall facility score was 72 (IQR 67–74; Table 1). Overall facility scores did not appear to differ by district (p = 0.11; Fig 1a).

**Table 1:** Balanced scorecard of health facilities overall and by district (N = 29).

Facility Scores	Health Facility Scores, % Median (IQR)									
	All		Chilanga		Chongwe		Kafue		Rufunsa	
	N = 29		N = 5		N = 12		N = 9		N = 3	
**Overall Facility Score**	72	(67–74)	78	(70–80)	72	(67–74)	73	(71–74)	65	(58–71)
**Patient Satisfaction Domain**	75	(71–78)	78	(77–78)	74	(69–79)	74	(73–75)	65	(60–79)
Patient satisfaction index—Children	75	(68–78)	80	(77–83)	72	(63–82)	75	(72–75)	58	(47–78)
Patient satisfaction index—Adults	75	(73–78)	75	(74–75)	75	(71–81)	75	(73–78)	73	(71–79)
**Human Resources Domain**	85	(63–87)	63	(62–88)	85	(83–86)	86	(62–90)	86	(84–89)
Health worker motivation score	73	(69–76)	76	(73–76)	70	(67–73)	75	(73–79)	71	(68–78)
Health worker training in past year	100	(50–100)	50	(50–100)	100	(0–100)	100	(50–100)	100	(100–100)
**Finance Domain**										
Finance index	50	(50–67)	75	(50–75)	50	(50–58)	50	(50–67)	50	(50–75)
**Governance Domain**										
Governance index	82	(74–91)	84	(81–93)	79	(68–85)	85	(81–93)	74	(72–90)
**Service Capacity Domain**	77	(68–79)	77	(77–79)	77	(69–82)	78	(76–79)	64	(51–72)
Basic infrastructure index	79	(75–83)	71	(67–79)	77	(71–83)	83	(79–83)	75	(46–75)
Basic equipment index	75	(70–95)	75	(70–95)	83	(68–98)	70	(70–90)	50	(30–80)
Laboratory capacity index	81	(63–81)	90	(63–100)	75	(65–81)	81	(63–81)	81	(38–86)
Tracer drugs index	81	(70–86)	81	(81–86)	71	(67–78)	90	(86–95)	67	(30–76)
Infection control index	72	(63–78)	67	(63–67)	78	(75–83)	56	(56–78)	67	(61–72)
**Service Provision Domain**	60	(52–76)	83	(78–83)	56	(48–64)	69	(58–77)	53	(34–60)
Service readiness index	75	(70–80)	85	(75–86)	75	(66–79)	73	(72–80)	76	(67–79)
Clinical observation index—Children	77	(60–84)	84	(77–85)	69	(59–80)	78	(71–95)	42	(15–84)
Clinical observation index—Adults	40	(0–60)	80	(60–80)	30	(0–40)	40	(20–80)	20	(0–60)

**Fig 2:**
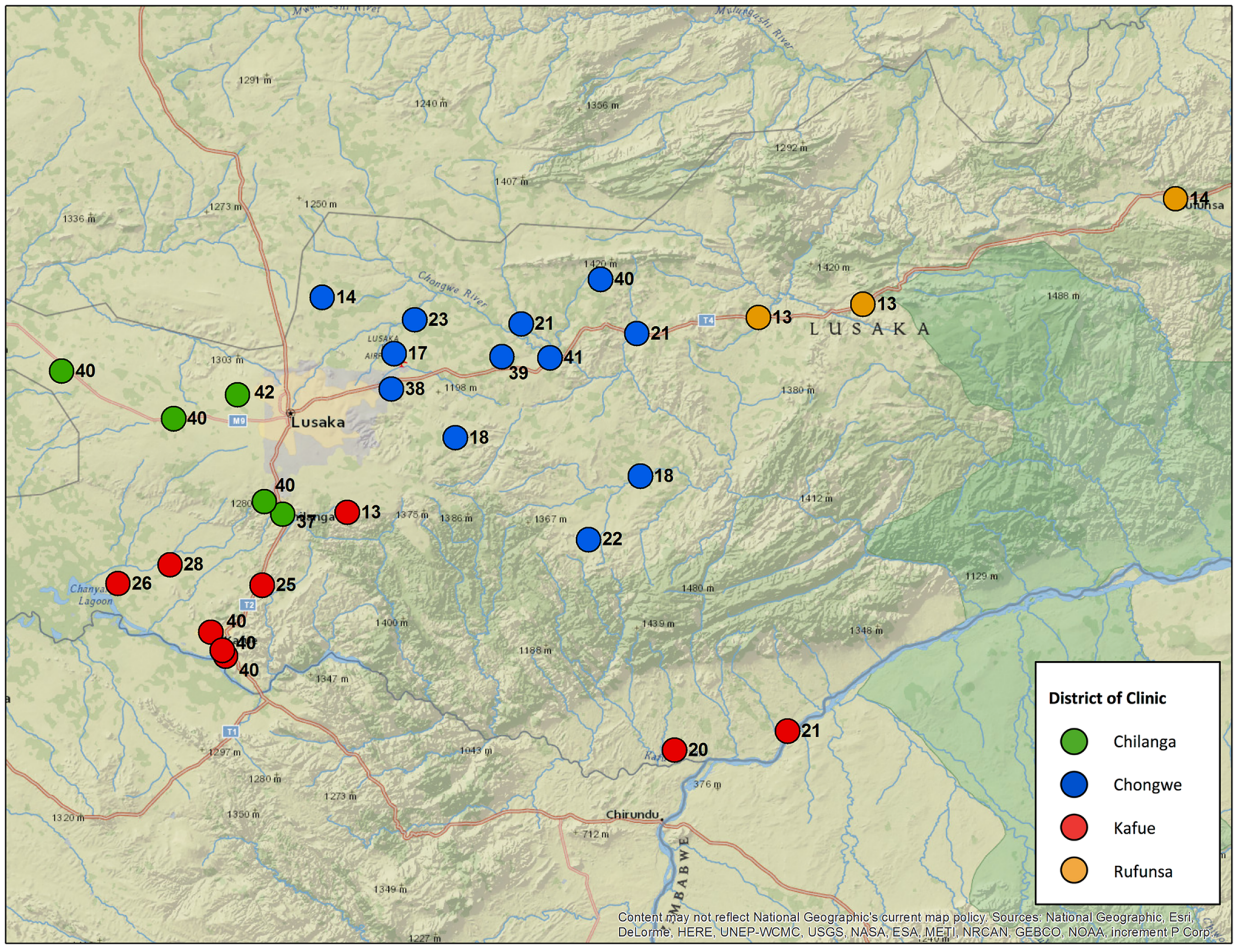
Participating clinics in Lusaka Province, Zambia with N representing number of mother-infant pairs enrolled at each clinic.

From June 2014 to November 2015, we enrolled HIV-infected mothers and their newborn infants into the community cohort component of SUVA. Across the 29 sites, we enrolled a total of 804 mother-infant pairs. The median number of mother-infant pairs enrolled at each site was 25 (IQR 18–40; Fig 2). Retention remained high throughout the 12-month SUVA follow-up period, with only 10 (1.2%) mother-infant pairs lost to follow-up or withdrawing early from the study. The overall proportion of reported antiretroviral drug use at 12 months postpartum was 80.7% (SD ±39.5%).

**Fig 3:**
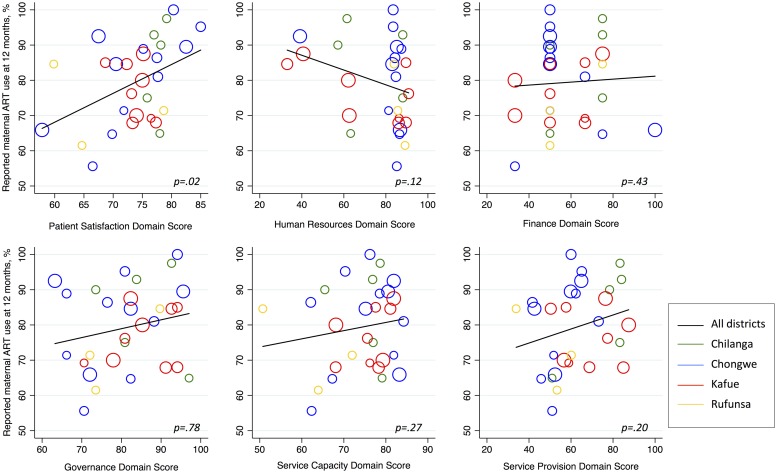
Scatter plot of reported 12-month postpartum maternal antiretroviral use by facility domain scores (N = 29).

Of the six domains, only patient satisfaction was significantly associated with our primary outcome (β = 0.22; 95%CI 0.04–0.41) in linear regression (Table 2). Other domains (i.e., finance, governance, service capacity, service provision) appeared to be positively associated with maternal antiretroviral drug use; however, none reached the threshold for statistical significance (Fig 3). Overall facility scores were not associated with reported 12-month maternal antiretroviral drug use.

**Table 2:** Linear regression of reported 12-month postpartum maternal antiretroviral use by facility overall and domain scores (N = 29).

Facility Score	Coefficient	95% CI	*p*
Patient Satisfaction	0.22	(0.04,0.41)	0.02
Human Resources	-0.41	(-0.94,0.12)	0.12
Finance	0.07	(-0.44,0.57)	0.78
Governance	0.18	(-0.15,0.50)	0.27
Service Capacity	0.10	(-0.16,0.36)	0.43
Service Provision	0.30	(-0.17,0.77)	0.20
Overall, all domains	0.09	(-0.10,0.28)	0.32
Composite 4 domains	0.18	(-0.03,0.40)	0.09
Chilanga + Chongwe, n = 17	0.24	(0.01,0.47)	0.04
Kafue + Rufunsa, n = 12	0.01	(-0.63,0.65)	0.97
Peri-urban facilities, n = 6	0.34	(0.19,0.48)	0.003
Rural facilities, n = 23	0.16	(-0.11,0.43)	0.28

**Fig 4:**
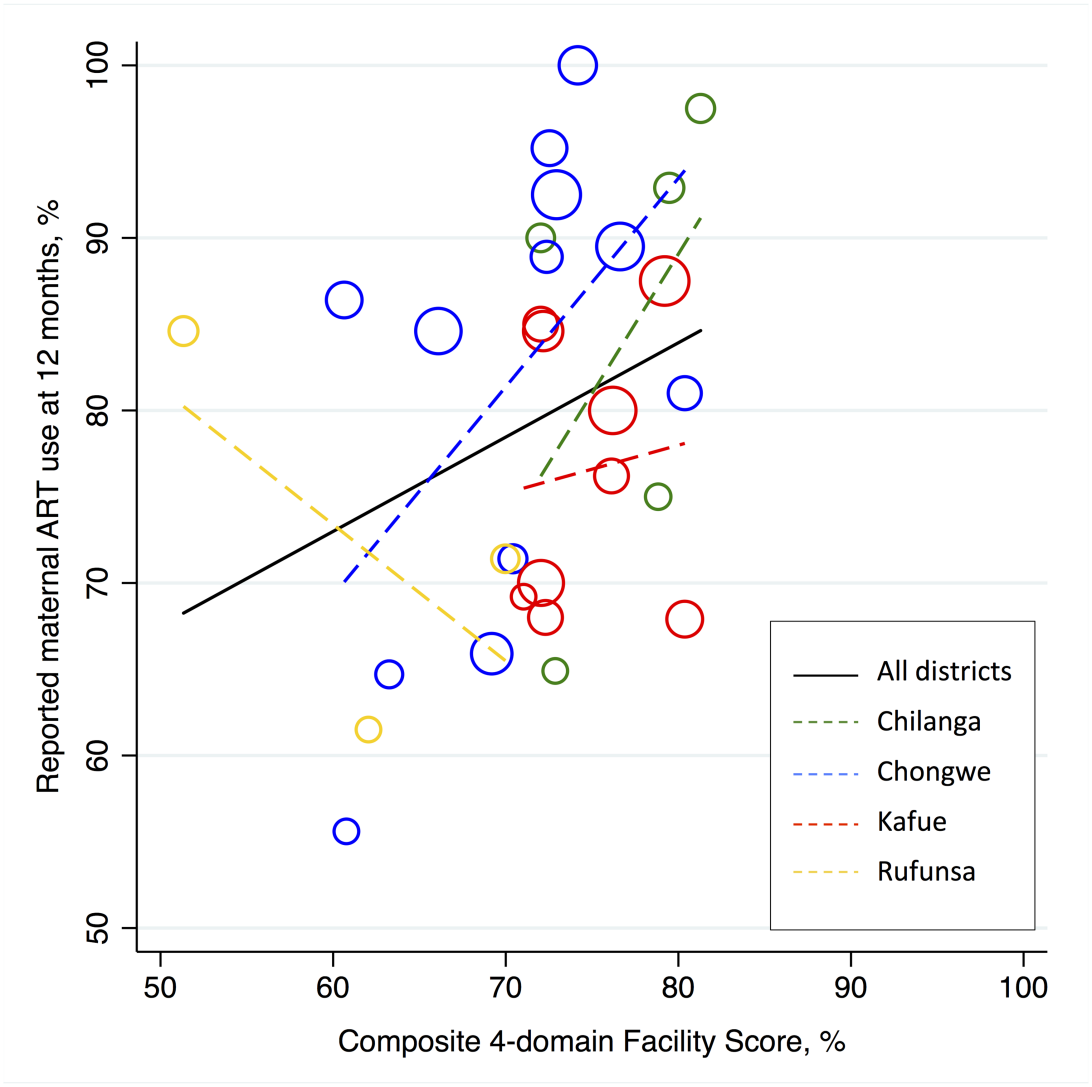
Scatter plot of reported 12-month postpartum maternal antiretroviral use by composite 4-domain facility score, stratified by district (N = 29).

In sensitivity analysis, we generated a composite 4-domain facility score by excluding the human resources and finance domains because of their non-normal distributions. These 4-domain facility scores—comprising patient satisfaction, governance, service capacity, and service provision—varied significantly by district (Fig 1b) and appeared to predict higher proportions of 12-month maternal antiretroviral drug use in some districts more than others (Fig 4). Composite 4-domain facility scores in Chilanga and Chongwe were positively associated with maternal antiretroviral drug use (β = 0.24; 95%CI 0.01–0.47), while those in Kafue and Rufunsa were not (β = 0.01; 95%CI -0.63–0.65). Similarly, facility scores in peri-urban facilities were associated with maternal antiretroviral drug use at 12 months (β = 0.34; 95%CI 0.19–0.48) in contrast to rural facilities (β = 0.16; 95%CI -0.11–0.43). The facilities with composite 4-domain facility scores in the lowest quartile had significantly lower proportion of maternal antiretroviral drug use at 12 months postpartum (median 69%, IQR 63–85) compared to those in the highest three quartiles (median 85%, IQR 71–90; *p* = .03).

## Discussion

The delivery of universal ART to pregnant and breastfeeding women requires strong health systems. To date, few studies have shown a relationship between health care capacity and programmatic outcomes at the site level. In this secondary analysis, we found several domain-specific scores showed positive correlation and one (patient satisfaction) met our *a priori* threshold for statistical significance. While our primary analysis showed no association between overall health system capacity and maternal ART use at 12 months postpartum, we found heterogeneity at the district level and among peri-urban versus rural facilities. These findings suggest that the separate components of health systems capacity—and other basic characteristics of the health facility—may relate differently to health service utilization and population outcomes. Understanding such relationships can help to guide the allocation of resources for building capacity more efficiently and effectively.

We graded the capacity of health facilities using a balanced scorecard approach previously adapted and validated by our study team[16, 21, 22]. This instrument is a mixed method evaluative tool that employs interviewer- and self-administered quantitative and qualitative assessments among a representative sample of observations at each facility. The components of the scorecard are based on the WHO building blocks of health systems, a framework originally developed to facilitate the allocation of investments in health system strengthening[17]. While the framework itself has limitations when used to quantify system-wide intervention impact, it remains the most universally employed and understood[23]. In our analysis, the human resources and finance domains each adopted more discrete rather than continuous distributions, likely due to limited variation in responses. Measurement approaches that establish broader distributions within these domains could facilitate more granular analyses and should be considered in future work.

By leveraging data from two studies performed concurrently at the same facilities, we had the unique opportunity to investigate associations between facility-level health system capacity scores and community-level maternal ART utilization[16, 18]. However, the two studies were not originally designed to complement each other and their temporal implementation did not perfectly overlap. It is conceivable that by not directly measuring the facility capacity and outcome simultaneously and by excluding some health facilities because of low SUVA recruitment, we may have introduced error. Such limitations would more likely lead to Type II error (i.e., failure to reject a false null hypothesis).

We chose postpartum maternal ART use as our primary outcome because it is a relatively proximal measure of program impact. While maternal ART use is not a direct health outcome, it is an important indicator of longitudinal follow-up in the context of universal ART and one that corresponds strongly with maternal and infant health[5, 24]. We note other potential limitations as well. As a self-reported indicator, our primary outcome may have been influenced by reporting, recall, and social desirability biases. Although our study was implemented during the nationwide transition to Option B+ services for PMTCT (i.e., universal ART for all pregnant and breastfeeding women), we were unable to verify maternal ART adherence using viral load, drug levels, or other modalities. While various self-reported ART adherence measures have been validated in other programmatic contexts[25–27], it is possible that residual biases persisted. Such biases inherent to self-reported outcomes generally result in an over-estimation of ART use and could lead to improper rejection of the null hypothesis if the biases are over-represented among attendees at facilities with higher capacity scores. Furthermore, we cannot confirm the generalizability of our results and specifically whether our estimate of ART use postpartum is indicative of the larger population. By investigating a single process indicator as our programmatic outcome, we may miss other important associations with health systems capacity. However, because the primary outcome of the SUVA study—infant HIV-free survival—demonstrated low variability across sites, we opted to investigate the association between facility capacity scores and an outcome with greater heterogeneity[13]. Future studies could consider other steps in the PMTCT cascade—e.g., antepartum HIV testing, maternal ART initiation and viral suppression, infant prophylaxis and HIV-free survival—to determine whether health systems capacity may relate differently to other health behaviors and outcomes.

Of the six domains of health capacity evaluated, only patient satisfaction significantly predicted maternal ART use at 12 months postpartum. Several studies have shown that patient-provider interactions and overall patient satisfaction can influence ART uptake and adherence, retention in HIV care, and even viral suppression[28–32]. In the multi-country PEARL study, for example, we observed a significant association between the antenatal care quality score and PMTCT coverage. There was also a positive trend between PMTCT coverage and patient satisfaction; however, this did not reach statistical significance (p = 0.06)[31]. A qualitative study among teen antenatal attendees in Limpopo Province, South Africa reported that client-counselor relationship during pretest counseling was a key determinant in HIV testing, and fear of poor treatment by care providers was a major factor in adherence to subsequent recommendations[33]. Another study found that long clinic wait times and negative interactions with providers were significant barriers to antenatal ART uptake in Uganda[30]. Finally, lack of privacy in antenatal clinics—particularly as it can lead to involuntary disclosure—has been reported as a barrier to engagement and retention in care[34, 35]. Interventions directed at improving the patient experience in PMTCT programs could help to close gaps left by systems and interventions focused primarily on programmatic performance and clinical outcomes[36, 37]. Health facility evaluations in this study were broadly implemented and not limited to PMTCT programs or stakeholders. Future studies could directly investigate the relationship between PMTCT-specific program acceptability, uptake, adherence, and retention. In addition, similar to our study, evaluations should consider both adult and children’s experiences with care in determining the quality of health services from patients’ perspectives.

Each facility domain may contribute differently to the equitable delivery of evidence-based interventions and towards impact along the PMTCT cascade[38, 39]. Overall facility capacity—and systems strengthening interventions—may lead to varied effects on health outcomes depending on underlying facility and patient characteristics. Indeed, one criticism of the WHO framework is that individual building blocks, each independent and weighted equally, obviate a holistic and context-specific assessment of facility or system quality[23]. These findings suggest a need for further investigation of the impact of baseline health systems capacity and strengthening interventions on PMTCT process indicators and patient-level outcomes. The current drive to strengthen health systems should be benchmarked against appropriate service delivery outcomes in order to demonstrate the value of such efforts and justify targeted donor support[17].

## Conclusions

Although we note positive trends between several domain and overall facility capacity scores, patient satisfaction most strongly predicted maternal antiretroviral use at 12 months after delivery. The association between overall health system capacity and maternal antiretroviral drug use was stronger in peri-urban compared to rural facilities. Our findings demonstrate the need for an improved understanding of the facility- and participant-level factors that truly affect PMTCT program impact and health outcomes to promote targeted investment in health system strengthening towards these priorities.

## Supporting information

S1 Dataset (CSV)
